# Effect of proactive personality on career adaptability of higher vocational college students: the mediating role of college experience

**DOI:** 10.3389/fpsyg.2024.1333677

**Published:** 2024-05-09

**Authors:** Min Fang, Runsheng Pan, Rongqi Ding, Zhijin Hou, Danni Wang

**Affiliations:** ^1^Department of Public Teaching, Zhejiang Institute of Economics and Trade, Hangzhou, China; ^2^Faculty of Psychology, Beijing Normal University, Beijing, China; ^3^Normal College, Jimei University, Xiamen, China

**Keywords:** higher vocational student, proactive personality, career adaptability, college experience, social cognitive career theory

## Abstract

For higher vocational students, the college stage is an important period in their career development, and the college experience plays an important role in the relationship between their proactive personality and career adaptability, which in turn has a significant impact on their future career development. From the perspective of social cognitive career theory and taking 476 vocational students as samples, this paper explores the mediating role of college experience between proactive personality and career adaptability of vocational college students. The college experience scale is revised for higher vocational students, and it is verified to have good reliability and validity. SPSS and Amos were used to conduct correlation analysis，and the PROCESS macro was used for mediating effect analysis. The results show that the college experience of vocational students plays a partial mediating role in the effect of proactive personality on career adaptability. This work innovatively uses social cognitive career theory to explore the role of college experience in the relationship between proactive personality and career adaptability among vocational students. The theoretical models are established and empirical verification is conducted, confirming that higher vocational students’ college experience can affect their career adaptability. These results provide empirical evidence for vocational colleges to improve the career guidance of college students, and intervention measures are proposed to enhance students’ career adaptability during school years, thus promoting their sustainable development.

## 1 Introduction

Because of the highly complex environment caused by informatization and globalization, today’s career development is highly unpredictable and uncertain (Bright and Pryor, 2005), leading to uncertainty in future career development for college students (Lechner et al., 2016). Regarding career development, a college student cannot guarantee that they will find a job that matches their major after graduation, and even if they can find such a job, they will not necessarily have it for life. Faced with this changing social environment, it is difficult to succeed in the workplace without strong adaptability. As the founder of career construction theory, Savickas proposed that its core element is career adaptability (CA), which refers to individuals’ readiness and resources to deal with current and upcoming career development tasks, career transformation, and career-related trauma (Savickas and Porfeli, 2012; Savickas, 2020). As an important concept in the career education of college students, CA is a physical and mental quality that students should have (Li et al., 2021) and is closely related to their future career development (Jiang, 2016; Rudolph et al., 2017). CA can not only increase the opportunities for individuals to find suitable jobs, but also help them to adapt successfully to all aspects of the workplace (Su et al., 2016). Overall, CA is considered to be an important prerequisite for career success (Koen et al., 2012), and can help college students to transition successfully into professionals (Maggiori et al., 2017).

With the advent of boundaryless careers, dynamic and continuous environmental changes have led to a more complex career environment, and researchers have also paid increasing attention to proactive personality (PP). Individuals in boundaryless careers should be more active in both career management and continuous lifelong learning (Jackson, 1996). Bateman and Crant (1993) proposed PP as a stable tendency and trend whereby individuals act to influence their external environment; i.e., individuals with PP are not easily affected by situational resistance but rather act actively to change their external environment. Some scholars have studied the relationship between PP and CA. Brown et al. (2006) found that individuals with PP are not only more successful in their career but also more able to adapt to their external environment. Li et al. (2013) showed that the higher the level of PP, the more actively individuals pay attention to their career development and the more they explore and attempt, and thus develop higher CA. McArdle et al. (2007) found that PP was significantly positively correlated with CA, and Cai et al. (2015) showed that self-esteem and PP positively predicted CA. Individuals with PP are more successful in developing their own CA resources than are individuals with inactive personality (Tolentino et al., 2014).

In the social cognitive career theory (SCCT) model of personal, environmental, and empirical factors that affect career-related choice behavior, learning experience is an important intermediate variable, one that plays a mediating role between personal and environmental variables and self-efficacy expectations and outcome expectations (Lent et al., 2013). Although the learning experience in SCCT differs in concept and perspective from the college experience (CE) in this study, it still has some similarities. In SCCT, learning experience is generally regarded by researchers as an important variable in individual career development. Although CE is only a part of learning experience, it is CE that can be concerned about and intervened with for a college. Teenagers are at an important stage of career exploration (Jiang et al., 2019), which helps individuals to clarify their future career development goals and make more-proactive career adaptation behaviors (Kaminsky and Behrend, 2015). Empirical research also indicates that career exploration positively predicts college students’ CA (Guan et al., 2015; Yang et al., 2021). Hu et al. (2021) noted that the college stage is the key period for students’ professional learning and the cultivation and development of various qualities and abilities, as well as being an important stage for career exploration. Hirschi (2009) showed that teenagers improve their CA through career exploration during school. Some research results have shown that there are significant differences in CA among groups of college students with different extents of participation in social practice activities: the more that college students participate in social practice activities, the higher their CA (Zhao, 2015).

The above literature review shows that CA and PP are attracting increasing research attention. However, although previous studies have shown that CE has a significant impact on career development (Akos et al., 2021), there has been little research to date on the relationship between CE and CA, especially the impact of specific dimensions of CE (e.g., professional learning experience, employment practice experience, and project learning experience) on CA. In particular, there is still a need for in-depth research—from theoretical models to CE scales to empirical verification—on the relationship among PP, CE, and career development abilities (such as CA).

The study reported herein is aimed at higher vocational students, who differ significantly from ordinary undergraduate students in that most vocational students begin working in society as soon as they graduate and have shorter school hours compared with ordinary undergraduates. They are relatively lacking in active self-planning consciousness, and most lack long-term goals and corresponding learning and development plans. Therefore, it is even more important to guide higher vocational college students to set goals, take positive actions, and use their limited school time and the resources around them efficiently in order to enhance their CA.

From the perspective of SCCT, this study explores further the mediating role of higher vocational students’ CE in the relationship between their PP and CA by establishing theoretical models and conducting empirical verification, based on exploring the impact of their PP on CA. In this study, because there is currently no readily available CE scale for vocational college students, the existing CE scale is revised and validated to support the present research. Based on these research results, this paper proposes intervention measures for vocational colleges on vocational students during their school years, thus better leveraging the role of CE in their career development and enhancing their CA.

### 1.1 Theoretical framework

Developed based on Bandura’s social cognitive theory, SCCT (Lent, 2013; Lent and Brown, 2019) is now applied mainly in the field of career development, being used to complement the basic theoretical methods of career development and to establish links among them. SCCT inherits the success of existing career theories, combines their highly similar elements as much as possible, and takes social cognitive theory as its main idea to integrate other relevant research results. SCCT combines personal characteristics, social background, and learning experience by virtue of submodels such as career interest, career choice, and job performance, and it discusses the process of career choice, adaptation, and development. It emphasizes the two-way and complex interaction among individuals, behaviors, and environment, and it points out that an individual’s unique learning experience plays an important role in determining and planning their career development path. SCCT is a relatively open theory, reasoning that the process from the formation of individual internal learning experience to the choice of careers is affected by various factors, which also provides various possibilities for effective intervention of career education in higher vocational colleges (Liu and Yao, 2015).

**Figure 1:**
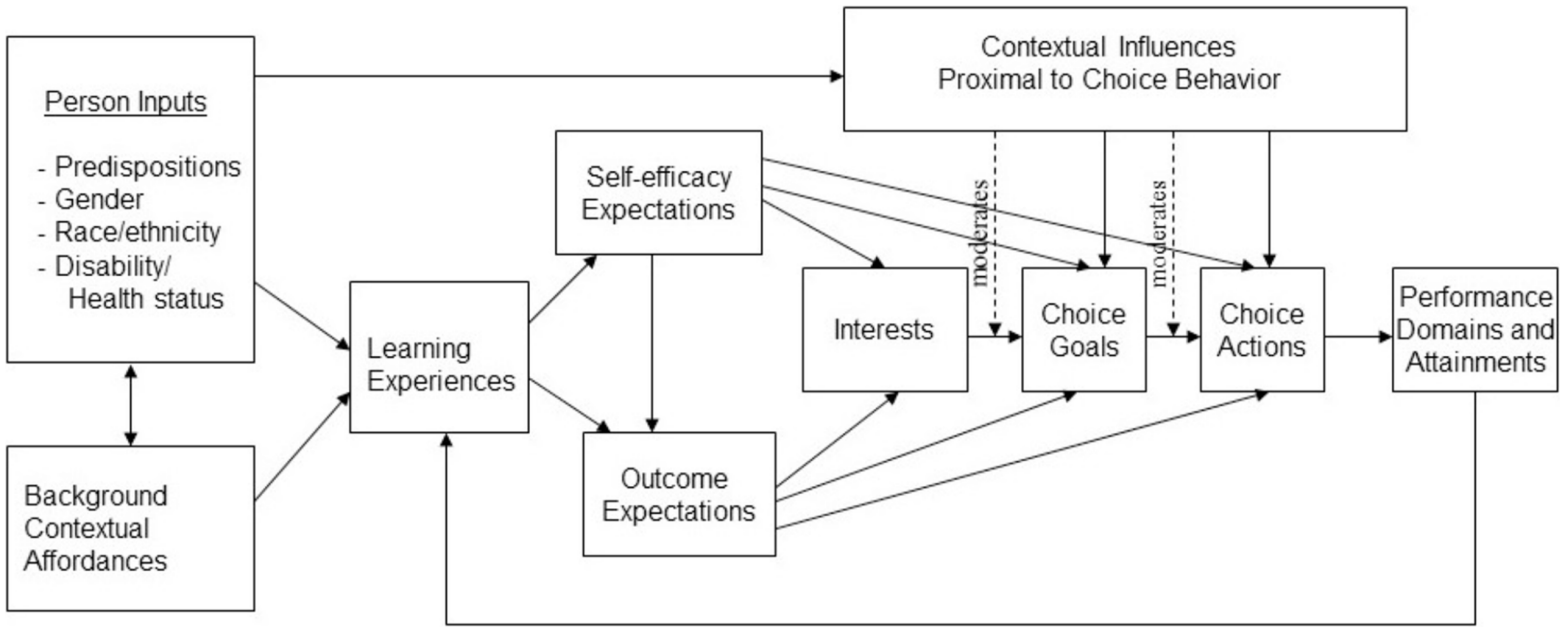
Theoretical model based on social cognitive career theory (SCCT). Solid lines correspond to direct relations between variables, and dashed lines correspond to moderator effects (where a given variable strengthens or weakens the relation between two other variables). Copyright 1993 by R.W. Lent, S.D. Brown, and G. Hackett. Reprinted by permission.

Based on SCCT (see Figure 1 for the specific theoretical model) (Lent and Brown, 2019), this study mainly explores the mediating role of the CE of vocational college students in the impact of PP on CA. PP is a personal predisposition that is part of Person Inputs, and CE is part of Learning Experiences, which influence both Self-efficacy Expectations and Outcome Expectations. In this way, CA is a part of Performance Domains that is continually shaped by an individual’s PP through various choices of CE.

### 1.2 Research hypotheses

Tolentino et al. (2014) showed that individuals with PP are not only more successful in their career but also more able to adapt to their environment. Some studies have found that college students’ PP can positively predict their CA level (Hou et al., 2014). Ling et al. (2022) showed that teenagers’ PP has a positive predictive effect on the CA of Chinese adolescents, i.e., their PP affects their future work self-significance and then affects their future time perspective and CA. Zhao et al. (2022)showed that PP has a positive impact on CA. According to these findings, Hypothesis 1 is proposed as follows.

*Hypothesis 1*: There is a significant positive correlation between the proactive personality and career adaptability of vocational college students.

Brown et al. (2006) showed that PP has a positive impact on career success, and individuals with strong CA are more likely to succeed in their career. Hirschi (2009) showed that the perceived social support of adolescents can enhance their CA during school. Hu et al. (2021) showed that college students with more PP can perceive more emotional support and work harder, thus improving their professional identity and CA. Based on these findings, Hypothesis 2 is proposed as follows.

*Hypothesis 2*: Higher vocational students’ college experience plays a mediating role in the effect of proactive personality on career adaptability.

**Figure 2:**
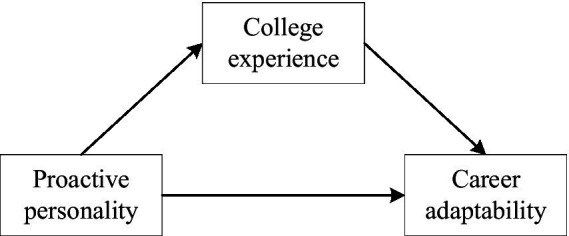
Hypothetical model of mediating role of college experience (CE) between proactive personality (PP) and career adaptability (CA).

Based on Hypothesis 1 and 2, the proposed hypothetical model is shown in Figure 2.

Regarding college students in mainland China, Zhao (2015) showed that their CA differed significantly with gender and grade but not with place of origin. Fu et al. (2022) showed that there are significant individual differences in the initial level of college students’ CA. Regarding high school students in Hong Kong, Leung et al. (2022) found that their CA differed significantly with gender and grade. As a further test of those dissimilarities in higher vocational students, Hypothesis 3 is proposed as follows.

*Hypothesis 3*: Career adaptability differs significantly with gender and grade.

Restubog et al. (2010) showed that the support of classmates and parents and the amount of career counseling are related to career self-efficacy and career decision-making. Zhao (2015) showed that in terms of the extent to which students participate in social practice activities, there are very significant differences in CA and its various dimensions among college students: the more that students participate in social practice activities, the higher their CA. College students are about to face the transition from school to work, which is also their first career transition. Active career exploration is an extremely important coping behavior that can help individuals to obtain relevant career information, accumulate more adaptive psychological resources, and finally achieve their career goals (Sonnentag et al., 2017).Other studies have found that training college students in vocational interests and skills can significantly promote individual career exploration (Owens et al., 2016). In view of this, Hypothesis 4 is proposed as follows.

*Hypothesis 4*: College experience differs significantly with grade.

## 2 Materials and methods

### 2.1 Participants and procedures

A prior power analysis was performed for sample size estimation by G*power 3.1. A sample size of 305 participants was needed for achieving a power of 0.95 to detect a medium effect size of *f* = 0.25 and an *α* level of 0.05.A convenience sampling method was used to select 570 vocational students from three ordinary colleges in Hangzhou and Shaoxing, which are convenient for research and are representative in Zhejiang Province. We had obtained permission from the participants before they completed the questionnaire during the class. SPSS 26.0 and Amos 24.0 were used for correlation analysis, and the PROCESS macro (Hayes and Scharkow, 2013; Hayes, 2018) was used for mediating effect analysis. We applied the criteria proposed by Hu and Bentler (1999) to evaluate the model fit: a combination of CFI > 0.90, TLI > 0.90, RMSEA <0.08, and SRMR <0.08 indicates a good model fit.

In these 570 copies of the questionnaire, 94 invalid ones were excluded, resulting in 476 valid ones. The survey sample includes 221 boys (46.4%) and 255 girls (53.6%), 195 of whom are freshmen (41.0%), 173 are sophomores (36.3), 108 are junior students (22.7%), 248 (52.1) of whom are science and engineering students, and 228 (47.9) are humanities students. In terms of age, 36 (7.6%) students are aged 18 to below, 217 (45.6%) are aged 19–20, 193 (40.5) are aged 21–22, and 30 (6.3%) are aged 23 and above.

### 2.2 Measures

#### 2.2.1 Selection of career adaptability and proactive personality scales

We used the Career Adapt-Abilities Scale–China Form (CAAS-China) revised by Hou et al. (2012). This scale involves 24 items, each of which describes an ability from “not strong” to “very strong” and scored on a five-point Likert scale; the higher the score, the stronger the degree of development of the ability. The items cover four dimensions: (i) career concern, (ii) career control, (iii) career curiosity, and (iv) career confidence. Each dimension involves six questions scored on a five-point Likert scale; the higher the score, the stronger the CA. After testing for internal consistency, the overall value of Cronbach’s alpha is 0.89, and the individual values for the four dimensions are between 0.64 and 0.79, indicating good reliability.

We also used the Proactive Personality Scale (PPS) developed by Bateman and Crant (1993) and revised by Shang and Gan (2009). This scale involves 11 items, each of which is scored on a seven-point Likert scale; 1 represents “strongly disagree” and 7 represents “strongly agree,” and the higher the score, the higher the PP. The retest reliability of the original scale is 0.72, and the internal consistency reliability is between 0.87 and 0.89. The internal consistency of the revised scale is 0.87.

#### 2.2.2 Revision of college experience scale

##### 2.2.2.1 Selection and revision of college experience scale

As for the CE scale, there is no ready-made CE scale for higher vocational students, so we referred to the CE scale compiled by Jin et al. (2013) and revised it into one suitable for higher vocational students. First, we interviewed 10 vocational students (including graduates and students in school) and 10 vocational college teachers engaged in student employment, and we used the first stage of the questionnaire to solicit their opinions on the language expression of the questionnaire. After comprehensive consideration, the original question bank of the scale was formed, which we divided into five dimensions. Second, to improve the content validity of the items of the scale, we invited experts in psychology to discuss together, interpret each item, and delete items with duplicate content. Third, based on the actual CE of higher vocational students during the school period, we added items such as “obtaining vocational qualification certificates related to your current major during the school period,” “participating in practical training (short for practical training of vocational skills),”and “participating in the employment practice (including post placement practice) provided by the school,” among others. Finally, through comprehensive interviews and previous studies, we determined 35 items, which are divided into five dimensions of the typical CE of vocational college students, including professional learning experience, social work experience, employment practice experience, social internship experience, and project learning experience. The specific explanations for each dimension are as follows.

1. Professional learning experience: refers to academic performance, performance of acquiring scholarship, and participation in disciplinary competitions such as vocational skills competitions and career planning competitions.
2. Social work experience: refers to experience mainly involving participation in student organizations and clubs, including experience in club activities, student cadres, class assistance, and volunteering.
3. Employment practice experience: refers to participation in employment practice organized by the school.
4. Social internship experience: refers to individual participation in paid social internships and part-time work.
5. Project learning experience: refers to involvement in employment and entrepreneurship platforms, projects, or activities organized or provided by the school.

##### 2.2.2.2 Reliability and validity of college experience scale

243 students from three vocational colleges were selected for the preliminary test. We conducted confirmatory factor analysis (CFA) on the CE scale. Because the scale was revised based on the relevant theories and studies mentioned above and has a theoretical structure, we conducted CFA directly on the scale with the Amos software (ver. 24.0) to analyze whether the items used conform to the theoretical construction. The fitting index demonstrated by this model (CFI = 0.88, TLI = 0.87, RMSEA = 0.08, SRMR = 0.06) does not meet the criteria proposed by Hu and Bentler (1999). To improve the model fit, we addressed the modification output of Amos by allowing a correlation between the residuals of item 1 (after hard study, all course grades ranked in the top 10 of the class) and item 2 [excellent in professional courses with a score of 85 points or above (maximum score is 100 points)], a correlation between the residuals of item 1 (after hard study, all course grades ranked in the top 10 of the class) and item 3 [excellent in elective courses with a score of 85 points or above (maximum score is 100 points)], between item 4 (obtaining comprehensive scholarships or scholarships during school) and item 5 (obtaining academic excellence scholarship during school), between item 8 (obtaining vocational qualification certificates related to your current major during school) and item 9 (vocational qualification certificates unrelated to your current major but related to future employment obtained during school), as well as between 21 (participation in summer social practice activities) and 22 (participation in social research activities). These correlations are reasonable, as item 1 and 2, as well as item 1 and 3, all involve course grades during school; item 4 and 5 both involve scholarships during school years; item 8 and 9 both involve professional qualification certificates; item 21 and 22 both involve social practice activities. According to Byrne et al. (1989), it is frequently needed to account for correlated errors to achieve a good model fit in structural equation modeling. Such adjustments are reasonable because they commonly reflect nonrandom measurement error stemming from factors like the item format similarity within a subscale. The results are given in Table 1. The model demonstrates improved and acceptable model fit indices. The analysis results show that the model’s fit is good, indicating that so is the validity structure of the scale.

**Table 1:** Fitting indices of confirmatory factor analysis of CE scale.

Fitting index	*χ* ^2^	*df*	*χ*^2^/*df*	TLI	CFI	RMSEA	SRMR
Value	1201.21	545	2.20^*^	0.90	0.91	0.07	0.06

(^*^p < 0.05).

Finally, we conducted reliability analysis on the CE scale. The scale involves 5 dimensions and totally 35 items, and each item is scored on a five-point Likert scale; the higher the score, the richer the CE.As given in Table 2, the value of Cronbach’s alpha for the scale is 0.97, indicating that the overall consistency reliability is relatively high. The values of Cronbach’s alpha for the various factors are 0.91, 0.88, 0.86, 0.86, and 0.96, respectively. After 2 weeks, 88 people were retested, and 52 valid copies of the questionnaire were received. The reliability analysis results show that the retest correlation coefficients of the five dimensions are 0.56, 0.70, 0.29, 0.63, and 0.61, respectively (see Table 2 for details), indicating that the retest reliability of the questionnaire is good.

**Table 2:** Reliability analysis of CE scale.

Dimension	Reliability	
	Internal consistency coefficient	Retest reliability (after 2 weeks)
	*N* = 243	*N* = 52
Professional learning	0.91	*r* = 0.56^**^
Social work	0.88	*r* = 0.70^**^
Employment practice	0.86	*r* = 0.29^*^
Social internship	0.86	*r* = 0.63^**^
Project learning	0.96	*r* = 0.61^**^
College experience scale	0.97	*r* = 0.46^**^

(^*^p < 0.05, ^**^p < 0.01).

## 3 Results

### 3.1 Sample descriptive statistics

#### 3.1.1 Career adaptability

The CA of higher vocational students and their scores in various dimensions are given in Table 3. As can be seen, the CA of the surveyed higher vocational students was generally at the upper middle level, with the highest score in the dimension of “career control” and the lowest in “career concern.” Independent-samples *t*-tests and analysis of variance (ANOVA) showed that the total score of CA did not differ significantly with gender, grade, and place of origin.

**Table 3:** Descriptive statistical results for CA.

	Career concern	Career control	Career curiosity	Career confidence	Career adaptability
*M*	3.56	3.88	3.60	3.66	3.72
SD	0.66	0.62	0.60	0.63	0.73

#### 3.1.2 Proactive personality

Analyzing the data for the PP of the surveyed higher vocational students showed that their PP status was generally in the upper middle level (*M* = 5.40, SD = 1.06), and independent-samples *t*-tests and ANOVA showed that their PP did not differ significantly with gender, grade, and place of origin.

#### 3.1.3 College experience

The CE scores of the surveyed higher vocational students are given in Table 4. As can be seen, their CE status was generally above the middle level, with the highest score in the dimension of “professional learning experience” and the lowest score in the dimension of “social work experience.” Independent-samples t-tests and ANOVA showed that their CE did not differ significantly with gender and place of origin but did differ significantly with grade. The LSD analysis of various dimensions showed that there were significant differences in professional learning between freshmen and sophomores (*p* < 0.01) as well as between freshmen and juniors (*p* < 0.01), in social work between freshmen and sophomores (*p* < 0.01) as well as between freshmen and juniors (*p* < 0.05), and in project learning between freshmen and sophomores (*p* < 0.01) as well as between freshmen and juniors (*p* < 0.01), while there were no significant differences in these dimensions between sophomores and juniors; however, employment practice differed significantly between any two of freshmen, sophomores and juniors (*p* < 0.01, *p* < 0.05), and social internship differed significantly between any two of freshmen, sophomores and juniors(*p* < 0.01). As shown in Table 5, gender differences in social work, employment practice, and project learning were significant, with males scoring higher than females.

**Table 4:** Descriptive statistical results for CE.

	Professional learning	Social work	Employment practice	Social internship	Project learning	College experience
*M*	2.36	1.89	2.12	2.32	2.06	3.64
SD	0.90	1.02	0.98	1.00	1.01	0.75

**Table 5:** Analysis of differences in various dimensions of CE by gender.

		Professional learning	Social work	Employment practice	Social internship	Project learning	College experience
Male	*M*	2.42	2.05	2.33	2.40	2.24	2.60
	SD	0.96	1.12	1.02	1.03	1.10	0.99
Female	*M*	2.31	1.75	1.93	2.25	1.90	2.47
	*SD*	0.84	0.90	0.91	0.96	0.90	0.92
*t* value		1.32	3.25^***^	4.49^***^	1.66	3.75^***^	1.52

^***^p < 0.001.

### 3.2 Correlation analysis

#### 3.2.1 Relationships among proactive personality, college experience, and career adaptability

To study further the relationships among the PP, CE, and CA of vocational college students, correlation analysis was conducted for each variable (see Table 6 for details). The results show that the CA of vocational college students is significantly positively correlated with their PP, which is as hypothesized and consistent with previous research results; furthermore, PP, CA, and CE are all significantly positively correlated.

**Table 6:** Mean, variance, and correlation coefficient of each variable (*N* = 476).

	*M*	*SD*	1	2
1. CA	3.72	0.73	–	
2. CE	3.64	0.75	0.25^**^	–
3. PP	2.53	0.95	0.28^**^	0.09^*^

(^*^p < 0.05, ^**^p < 0.01).

#### 3.2.2 Partial mediating role of college experience

**Figure 3:**
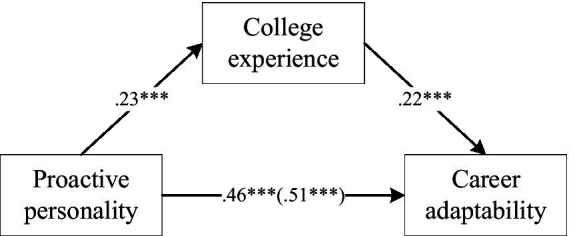
Mediating effect model of CE between PP and CA.

We explored the mediating effect of CE in the relationship between PP and CA (The analysis results are shown in Tables 7, 8). The test analysis was conducted based on controlling for gender, grade, and place of origin, with PP as the independent variable, CA as the dependent variable, and CE as the mediating variable. The result showed that the indirect effect of CE [0.03, 95% CI = (0.02, 0.05)] explains 9.60% of the total effect [0.30, 95%CI = (0.26, 0.35)]. This indicates that CE plays a mediating role between PP and CA, which supports Hypothesis 2 (see Figure 3).

**Table 7:** Test of mediating model of CE (*N* = 476).

Outcome variable	Predictive variables	*R*	*R* ^2^	*F*	*β*	*SE*	*t*
CA		0.52	0.27	42.73			
	Gender				0.04	0.04	0.89
	Grade				0.03	0.03	0.76
	Place of origin				−0.07	0.02	−1.67
	PP				0.51	0.02	12.88^***^
CE		0.42	0.17	24.81			
	Gender				−0.09	0.07	−2.23^*^
	Grade				0.31	0.04	7.22^***^
	Place of origin				−0.04	0.03	−0.91
	PP				0.23	0.04	5.47^***^
CA		0.55	0.31	41.15			
	Gender				0.06	0.04	1.43
	Grade				−0.04	0.03	−0.86
	Place of origin				−0.06	0.02	−1.50
	PP				0.46	0.02	11.57^***^
	CE				0.22	0.03	5.08^***^

^*^p < 0.05; ^***^p < 0.001. Using proactive personality as the independent variable, college experience as the mediating variable, career adaptability as the dependent variable, and gender, grade, and place of origin as control variables, SPSS with the PROCESS macro was used to test the mediating effect, and the mediating effect was significant.

**Table 8:** Decomposition table of total effect, direct effect, and mediating effect.

	Effect value	Boot SE	Boot LLCI	Boot ULCI	Relative effect value
Total effect	0.30	0.02	0.26	0.35	
Direct effect	0.27	0.02	0.23	0.32	90.40%
Mediating effect	0.03	0.01	0.02	0.05	9.60%

The boot standard error, boot CI lower limit, and boot CI upper limit refer to the standard error, 95% confidence interval lower limit, and upper limit of the indirect effects estimated using the bias corrected percentile bootstrap method, respectively; all values are rounded to 2 decimal digits.

## 4 Discussion

### 4.1 Findings

Main findings of this study are summarized as follows. First, the CA and PP of the vocational college students were generally at the upper middle level. The research results showed that as hypothesized, their PP and CA had a significant positive correlation. Second, in terms of CA, “career control” scored the highest, followed by “career curiosity,” “career confidence,” and “career concern.” CA did not differ significantly with gender, grade, or place of origin, which is not as hypothesized. Third, the research results showed that as hypothesized, CE plays a partial mediating role between the PP and CA of vocational college students. Fourth, it was found that the CE of vocational college students is generally at the upper middle level, with the highest score in the dimension of “professional learning experience” and the lowest score in the dimension of “social work experience.” CE does not differ significantly with gender or place of origin. The effect of grade is reflected mainly in the differences between freshmen and sophomores/juniors, and the differences between sophomores and juniors are not significant, which are not completely as hypothesized.

#### 4.1.1 College experience and its dimensions and demographic variables

This study showed that the level of the CE of the surveyed vocational college students was generally above the middle level, with the highest score in the dimension of “professional learning experience,” followed by “social internship experience,” “employment practice experience,” “project learning experience,” and “social work experience.” The low score of social work experience is due to the limited opportunities and platforms for students to participate in social work, as well as due to the lack of relevant systematic management mechanisms, resulting in a lack of social work experience for the vast majority of students. The low score in the dimension of “project learning experience” is due to the insufficient forms of employment and entrepreneurship guidance organized or provided by the college. Currently, vocational colleges focus mainly on only employment and entrepreneurship guidance courses, supplemented by job fairs, employment and entrepreneurship guidance lectures, and innovation and entrepreneurship competitions, and they lack systematic career group guidance, experiential career guidance activities, and personalized career consulting services. At the same time, some students have already received similar course content in high school, resulting in low participation enthusiasm.

Regarding demographic variables, CE did not differ significantly with gender and place of origin. In terms of grade, the differences between freshmen and sophomores or between freshmen and juniors, while the differences between sophomores and juniors were not significant, which are not entirely consistent with our hypothesis. When students enter the college as freshmen, they have just come into contact with college studies. Therefore, CE differs significantly with grade. On the other hand, some majors in higher vocational education are two-year ones, or students face graduation internships and other related matters in their junior year and the related activities in school are mainly completed before their sophomore year, resulting in an insignificant difference between sophomores and juniors.

#### 4.1.2 Mediating role of college experience

We found that the mediating effect was significant, but the mediating effect value was low. We believe that first, CE is likely to rely mainly on opportunities provided by higher vocational colleges. For example, people with high PP may have very little CE, not because they do not want to have these experiences, but because they do not have the opportunity to have these experiences. This also requires higher vocational colleges to provide more platforms and opportunities for vocational college students to enhance their CA through their CE. Second, we believe that PP is not the only influencing factor for CE and may be influenced by other factors such as career social support, but the results of this study at least partially explain the effect. Third, we need to further optimize the dimensions of the CE scale, for instance adding dimensions such as club activity experience based on the original 5 dimensions.

### 4.2 Implications

The theoretical significance of this study lies in revealing the mediating role of CE in the influence of PP on CA, and deepening the mechanism by which PP influences CA. This study innovatively used SCCT to explore the mediating role of CE in the relationship between PP and CA among vocational college students. The theoretical models were established and empirical verification was conducted, confirming that higher vocational students’ CE can affect their CA.

In practice, our findings provide effective reference for career education in higher vocational colleges, and we make the following suggestions.

First, the research results show that there is room for vocational college students to improve their CA. The score of the “career concern” dimension is the lowest, which indicates that vocational college students are relatively lacking in the ability of independent learning and the awareness of independent self-planning, and most of them have neither goals nor corresponding academic planning and development planning. On one hand, this requires higher vocational colleges to integrate the content of CA into their career education to inspire students to establish life design thinking and improve their CA. On the other hand, this requires higher vocational colleges to establish a three-level progressive-goal education model of “goal setting—goal implementation—goal assessment and evaluation” to guide students to independently plan their studies and establish a scientific and flexible goal management mechanism. Furthermore, we also need to build an integrated career education system for primary school, junior high school, senior high school and college.

Second, the results of this study show that higher vocational students lack social work experience. Therefore, vocational colleges should deepen the collaborative education of “college, government, administration, and enterprise” and improve the practicality of career guidance. For instance, vocational colleges could implement career education models such as “one-on-one guidance of career mentors” and “career mentors entering the classroom.” On one hand, vocational colleges should encourage teachers to connect with industry enterprises, make full use of school and government resources, introduce real enterprise projects into the school, lead student teams to participate in enterprise projects, and promote students’ growth in the process of “learning to do, learning by doing.” On the other hand, vocational colleges should arrange practical training and participate in enterprise projects and other activities in a planned, step-by-step, hierarchical, and classified way.

Finally, the research results show that vocational college students have differences in their CE and various dimensions, indicating that vocational colleges need to strengthen personalized career guidance. Higher vocational colleges should not only strive to balance the needs of enterprises and students and change the main position of teaching from public compulsory courses to public elective courses, but also set up group counseling courses with the themes of “improving CA,” “improving self-efficacy,” and “improving job skills” that students focus on. In addition, vocational colleges should integrate the resources of “schools, governments, industries, and enterprises,” create career group counseling courses and individual counseling platforms, and work together to pay attention to and effectively help the groups with employment difficulties.

### 4.3 Limitations and future research directions

This study had the following limitations. First, social desirability may have affected the subjects’ reports. They were asked to report their awards, professional achievements, etc., and their reports were easily disturbed by social expectations. Therefore, in data investigation, we should try to reduce this impact, for example by repeatedly emphasizing anonymity and academic significance and ensuring that their personal information will not be disclosed. Second, this study was a correlational study and so could not fully explain the causal relationship between variables. In the future, longitudinal study design could be conducted to clarify the causal relationship between variables.

Our future research directions include the following. First, we will expand the representativeness of the sample. In this study, the vocational college students were all from Zhejiang Province, and their homogeneity were relatively strong. In the future, we will expand the sample to other provinces to enhance the heterogeneity. Second, we will establish appropriate intervention programs to enhance the CA of vocational college students. Third, we can further optimize the dimensions of the CE scale, for instance adding dimensions such as club activity experience based on the original 5 dimensions.

## 5 Conclusion

This study explores the role of vocational college students’ CE in the relationship between their PP and CA from the perspective of social cognitive career theory. The results indicate that CE plays a mediating role between PP and CA. Based on these research results, this paper proposes intervention measures for vocational colleges on vocational students during their school years, thus better leveraging the role of CE in their career development and enhancing their CA.

## Data availability statement

The raw data supporting the conclusions of this article will be made available by the authors, without undue reservation.

## Ethics statement

The studies involving humans were approved by Scientific Research Office, Zhejiang Institute of Economics and Trade. The studies were conducted in accordance with the local legislation and institutional requirements. The participants provided their written informed consent to participate in this study.

## Author contributions

MF: Conceptualization, Data curation, Formal analysis, Funding acquisition, Investigation, Methodology, Resources, Software, Validation, Visualization, Writing – original draft. RP: Formal analysis, Writing – review & editing. RD: Formal analysis, Writing – review & editing. ZH: Conceptualization, Project administration, Supervision, Writing – review & editing. DW: Writing – review & editing.
